# Artificial Intelligence-Guided Personalized Gut Microbiome Modulation for Persistent Secondary Gastrointestinal Symptoms in Oncology Patients: Clinical Efficacy and Biological Correlates from a Prospective Validation Study

**DOI:** 10.3390/cancers18091453

**Published:** 2026-05-01

**Authors:** Radu Dumitru Dragomir, Sorin Saftescu, Daniela Lidia Sandu, Ana Dulan, Irina Mihaela Croitoru-Cazacu, Adina Emilia Croitoru, Vlad Mihai Croitoru, Vlad Vornicu, Daniela Elena Nagy, Iulia Teodora Perva, Diana Sirca, Dorel Ionel Popovici

**Affiliations:** 1 Department of Oncology, Faculty of Medicine, “Victor Babes” University of Medicine and Pharmacy, Eftimie Murgu Square 2, 300041 Timisoara, Romania; radu.dragomir@umft.ro (R.D.D.); daniela.sandu@umft.ro (D.L.S.); ana.dulan@umft.ro (A.D.); dorel.popovici@umft.ro (D.I.P.); 2 Department of Oncology, Fundeni Clinical Institute, Carol Davila University of Medicine and Pharmacy, 022328 Bucharest, Romania; irina-mihaela.cazacu@umfcd.ro (I.M.C.-C.); adina.croitoru@umfcd.ro (A.E.C.); vlad.m.croitoru@gmail.com (V.M.C.); 3 Doctoral School in Medicine, “Victor Babes” University of Medicine and Pharmacy, 300041 Timisoara, Romania; vlad.vornicu@umft.ro; 4 OncoHelp Oncology Center, 300239 Timisoara, Romania; dana.nagy85@gmail.com (D.E.N.); iulia.perva@umft.ro (I.T.P.); 5 Faculty of Medicine, “Victor Babes” University of Medicine and Pharmacy, 300041 Timisoara, Romania; diana-cristiana.sirca@student.umft.ro

**Keywords:** cancer survivorship, gastrointestinal toxicity, gut microbiome, dysbiosis, personalized medicine, artificial intelligence, microbiome modulation, short-chain fatty acids, *Bifidobacterium longum*, precision supportive care

## Abstract

**Background/Objectives**: Persistent gastrointestinal (GI) symptoms following oncologic treatment represent a major unmet need in survivorship care, often managed symptomatically without addressing underlying biological mechanisms. This study aimed to evaluate the clinical efficacy and biological correlates of an artificial intelligence (AI)-guided, personalized microbiome modulation strategy in oncology patients with chronic secondary GI dysfunction. **Methods**: We conducted a prospective, single-arm, open-label validation study including 29 adult female oncology patients with persistent GI symptoms lasting ≥3 months. Participants underwent baseline multidimensional assessment integrating shotgun metagenomic sequencing, inflammatory and nutritional biomarkers, and clinical symptom profiling. An AI-guided platform generated individualized dietary, supplement, and lifestyle recommendations. Outcomes were assessed at baseline and after a 3-month intervention, focusing on intra-individual changes in stool frequency (primary endpoint), self-reported energy, microbiome composition, and metabolic biomarkers. Paired statistical analyses, correlation testing, and multivariable regression were performed. **Results**: After three months, stool frequency significantly decreased (4.69 ± 2.41 to 2.07 ± 1.19 episodes/day; *p* < 0.0001), accompanied by a marked increase in energy levels (4.00 ± 1.04 to 7.24 ± 1.12; *p* < 0.0001). Microbiome analysis revealed consistent enrichment of butyrate-producing and barrier-supportive taxa, including *Faecalibacterium prausnitzii*, *Eubacterium rectale*, *Roseburia intestinalis*, *Akkermansia muciniphila*, and *Bifidobacterium longum*. Butyrate-related biomarkers and vitamin-associated parameters (B-complex, vitamin D) showed significant improvement, while lactate levels normalized. Changes in *Bifidobacterium longum* were independently associated with stool frequency reduction (β = −0.783, *p* = 0.0082). **Conclusions**: AI-guided personalized microbiome modulation was associated with significant clinical improvement and biologically coherent microbial and metabolic shifts in oncology patients with persistent GI symptoms. These findings support a precision supportive-care approach targeting microbiome restoration, warranting further validation in randomized controlled trials.

## Simple Summary

Many cancer patients continue to experience long-lasting digestive problems such as diarrhea, bloating, and abdominal discomfort even after completing treatment. These symptoms are often managed with general measures that do not address the underlying cause, which may involve an imbalance in the gut microbiome. In this study, we evaluated a personalized approach that uses artificial intelligence to analyze each patient’s microbiome, clinical data, and laboratory results in order to generate individualized dietary and supplement recommendations. After three months, most patients showed clear improvement in bowel habits and overall energy levels. These clinical changes were accompanied by favorable changes in beneficial gut bacteria and related metabolic markers. Our findings suggest that personalized microbiome modulation may represent a promising strategy for improving long-term quality of life in cancer survivors and highlight the potential role of data-driven approaches in supportive oncology care.

## 1. Introduction

Over the past decades, progress in oncology has transformed many cancers from rapidly fatal diseases into chronic conditions. Increasingly effective chemotherapy regimens, immunotherapy, targeted agents, and multimodal treatment strategies have extended survival across a wide range of malignancies [1]. Yet, as survival improves, a new clinical reality has emerged: many patients live for years with persistent treatment-related toxicities that significantly affect daily functioning. Among these, chronic gastrointestinal (GI) symptoms remain some of the most frequent and distressing complaints encountered in routine oncologic practice [2,3].

In clinical settings, patients frequently report ongoing diarrhea, constipation, bloating, abdominal discomfort, excessive gas, and food intolerance long after the acute phase of treatment has ended [4]. These symptoms are often temporally associated with chemotherapy, radiotherapy, immunotherapy, corticosteroid exposure, or repeated antibiotic courses [5]. While rarely life-threatening, they substantially impair quality of life, nutritional status, treatment adherence, and psychological well-being. Importantly, current management strategies are predominantly symptomatic (antidiarrheals, laxatives, empirical probiotics, dietary exclusions) without addressing the underlying biological disturbances that may perpetuate these manifestations [6].

In recent years, growing attention has been directed toward the intestinal microbiome as a key mediator of both cancer treatment response and toxicity [7]. Antineoplastic therapies can profoundly disrupt microbial ecosystems, reducing diversity, altering taxonomic composition, and modifying microbial metabolic output. Such dysbiosis may compromise epithelial barrier integrity, promote immune dysregulation, and sustain low-grade inflammation [8]. In some patients, this altered microbial state appears to persist beyond the completion of oncologic therapy, potentially contributing to chronic gastrointestinal dysfunction [9]. Despite this increasing recognition, microbiome-targeted interventions in oncology have largely remained empirical and non-individualized.

One of the major challenges in translating microbiome science into clinical practice lies in its complexity. Microbial communities interact dynamically with host metabolism, immune pathways, nutritional status, cancer type, and treatment modality. A standardized, “one-size-fits-all” approach is unlikely to capture this biological heterogeneity [10]. Advances in high-throughput sequencing technologies and data science now allow for integration of microbiome profiling with laboratory biomarkers and detailed clinical phenotyping. Artificial intelligence (AI) based analytical platforms provide the capacity to process these multidimensional datasets and generate personalized, biologically informed recommendations [11,12].

The present study was conceived from a practical clinical need: to move beyond symptomatic management and evaluate whether an AI-guided, personalized microbiome modulation strategy could meaningfully improve persistent secondary GI symptoms in oncology patients [13]. By integrating baseline shotgun metagenomic sequencing, inflammatory and nutritional biomarkers, detailed medical history, and patient-reported outcomes, the AI-guided platform generated individualized dietary and supplement-based recommendations using commercially available products. The objective was not only to evaluate symptom evolution over a three-month period, but also to explore potential biological correlates of clinical symptom response.

By examining both clinical efficacy and microbiome-related dynamics, this study seeks to contribute to the emerging field of precision supportive oncology. Addressing chronic gastrointestinal toxicity is essential not merely for symptom control, but for restoring functional recovery and improving long-term survivorship outcomes in patients who have already endured complex oncologic treatments [14].

## 2. Materials and Methods

### 2.1. Study Design and Setting

This study was conceived as a prospective, single-arm, open-label validation project aimed at evaluating whether an AI-guided personalized microbiome modulation strategy could meaningfully improve persistent secondary gastrointestinal symptoms in oncology patients. Rather than comparing against a control group, the primary objective was to assess intra-individual change over time and to explore the biological coherence between microbiome shifts and symptom evolution. In this context, a single-arm design was considered appropriate, as each patient effectively served as their own control through paired baseline and follow-up assessments.

The study followed a decentralized structure and included a three-month active intervention period for each participant. Clinical, laboratory, and microbiome evaluations were performed at baseline (Month 0) and repeated at Month 3. The emphasis was placed on capturing real-world dynamics in a supportive-care setting, without altering standard oncologic treatment. The follow-up duration was limited to three months, reflecting the initial validation design of the study.

The study was conducted in accordance with the Declaration of Helsinki and approved by the Ethics Committee of the OncoHelp Oncology Center (258/11 February 2025) for studies involving humans.

### 2.2. Study Population

Participants were adult oncology patients presenting with persistent gastrointestinal symptoms that developed in temporal association with cancer diagnosis or oncologic therapy.

Eligibility required histologically or clinically confirmed solid or hematologic malignancy, age ≥18 years, and the presence of chronic secondary GI symptoms for at least three months. These symptoms included diarrhea, constipation, bloating, abdominal discomfort, or pain. To minimize confounding variability, only patients on stable oncologic regimens for at least four weeks prior to enrollment were included. The inclusion criterion was based on symptom persistence rather than severity, which resulted in a heterogeneous baseline clinical profile. This reflects real-world clinical practice, where patients may present with varying degrees of gastrointestinal dysfunction despite similar symptom duration.

Patients were excluded if they had active gastrointestinal infections, primary inflammatory bowel disease, celiac disease, recent antibiotic exposure within four weeks before baseline sampling, known intolerance to potential supplement components, or any medical instability requiring urgent intervention.

Importantly, the intervention was conceived as an adjunct supportive-care strategy. Ongoing cancer treatments were neither modified nor delayed during participation in the study. All patients continued routine oncologic follow-up throughout the three-month period.

The cohort consisted exclusively of female participants and was relatively young, reflecting the recruitment pathway, which predominantly included breast and gynecologic cancer survivors. This limits the generalizability of the findings, as sex- and age-related biological differences may influence microbiome composition, immune responses, and clinical outcomes. Consequently, the observed effects may not be directly applicable to male patients or to older oncology populations with different comorbidity profiles and treatment exposures. Future studies including more diverse and representative cohorts are necessary to validate the external applicability of these findings.

### 2.3. Baseline Multidimensional Assessment

At enrollment, each participant underwent a structured and multidimensional baseline evaluation. The aim was not only to document symptom burden but to characterize the biological context in which those symptoms persisted.

Stool samples were collected for baseline shotgun metagenomic sequencing to assess taxonomic composition and functional microbial pathways. Microbial abundance was reported using predefined ordinal laboratory reference categories (ranging from very low to very high), which later allowed paired directional analysis between baseline and follow-up.

Blood samples were obtained to evaluate inflammatory and nutritional biomarkers relevant to gastrointestinal integrity and systemic recovery. A comprehensive review of medical records was performed, documenting cancer type, treatment modality, and symptom chronology.

Patients also completed structured questionnaires assessing gastrointestinal symptom patterns, dietary habits, and overall well-being. A daily stool diary was initiated at baseline and maintained throughout the intervention period, allowing continuous documentation of bowel frequency and symptom fluctuation.

Microbiome data were provided by the laboratory platform in the form of predefined ordinal abundance categories (very low to very high), rather than continuous quantitative metagenomic counts. Raw sequencing data (e.g., read counts or relative abundance percentages) were not directly accessible for independent analysis. This approach reflects the reporting framework of the clinical microbiome platform used in this study. It should be noted that for some biomarkers, the clinical interpretation of directional change may differ from ordinal category direction (e.g., reduction in lactate levels representing improvement).

This integrative baseline profiling formed the foundation for the individualized microbiome modulation strategy applied in the subsequent three months.

### 2.4. AI-Guided Personalized Microbiome Modulation

The AI-guided analytical platform was designed to integrate multidimensional host–microbiome data within a dynamic systems-level framework, moving beyond static taxonomic interpretation toward a functional and context-aware understanding of gastrointestinal dysregulation. Rather than analyzing microbial composition in isolation, the platform incorporates a layered model in which microbiome features are interpreted alongside inflammatory biomarkers, metabolic parameters, nutritional status, and clinical symptom profiles.

The primary input variables include microbiome composition derived from metagenomic sequencing, inflammatory and nutritional biomarkers, clinical history, and patient-reported symptom profiles.

To enhance transparency and reproducibility, the AI system used in this study follows a hybrid architecture combining a curated microbiome-host interaction knowledge graph with a deterministic rule-based inference engine, rather than a purely data-driven black-box machine-learning model. This distinction is essential, as the system does not rely on parameter training on labeled outcome datasets but instead on structured integration of literature-derived biological relationships and predefined clinical rules.

A central component of the analytical framework is the identification of individualized dysbiosis signatures based on the interaction between microbial communities and host-derived signals. In contrast to conventional approaches that rely on relative abundance thresholds, the platform evaluates the functional relevance of microbial patterns by contextualizing them within a dynamic inflammatory and metabolic environment. This allows the detection of clinically meaningful configurations such as impaired short-chain fatty acid production, pro-inflammatory microbial dominance, disrupted epithelial barrier support, and altered microbial–host metabolic coupling.

The computational engine integrates a rule-based inference framework with elements of machine-learning-assisted pattern recognition (e.g., gradient-boosted decision trees) embedded within a broader knowledge-graph architecture. Importantly, these components do not function as standalone predictive models trained on labeled outcome datasets but rather support structured interpretation of complex host–microbiome relationships.

Importantly, the concept of a “training dataset” in the conventional supervised machine-learning sense is not directly applicable to this system. Instead, the analytical framework is based on a continuously curated knowledge base derived from peer-reviewed literature, public biological databases, and clinical guidelines.

The system was developed through iterative curation of the knowledge base and clinical rule library, integrating evidence from peer-reviewed literature, biological databases, and clinical expertise, rather than through parameter training on labeled datasets.

A distinctive feature of the platform is its ability to incorporate dynamic inflammatory context into microbiome interpretation. Inflammatory markers are not treated as independent variables, but as modulators of microbial function and host susceptibility, enabling the algorithm to prioritize microbial alterations that are most likely to contribute to symptom persistence. This integrative approach allows the identification of actionable targets that reflect both microbial imbalance and host physiological state.

Unlike conventional machine-learning models, feature importance is not expressed as statistical weights but rather as traceable rule activation. Each recommendation generated by the system can be linked to specific microbial features, clinical parameters, and underlying literature sources, enabling full interpretability at the level of individual decision pathways.

Based on this multilevel analysis, the platform generates personalized therapeutic recommendations aimed at restoring ecological balance and improving gastrointestinal function. These recommendations may include targeted dietary interventions, selective use of prebiotic and probiotic compounds, correction of micronutrient deficiencies, and lifestyle modifications. Importantly, the intervention strategy is adaptive and individualized, reflecting the specific biological profile of each patient rather than applying standardized protocols.

System validation was performed at multiple levels. At the computational level, internal consistency checks and rule-based validation procedures were applied to ensure logical coherence and reproducibility of outputs across identical input profiles. At the clinical level, the system’s recommendations were evaluated through prospective application in patient cohorts, including the present study, focusing on clinical outcome coherence rather than conventional predictive performance metrics such as AUC or accuracy, which are not applicable to rule-based systems.

The input–output pipeline follows a structured sequence: (i) acquisition of patient-specific multi-omic and clinical data, (ii) extraction of microbial and host-derived functional signatures, (iii) mapping of these features onto the knowledge graph, (iv) application of deterministic clinical rules, and (v) generation of ranked, clinically actionable recommendations subject to physician review.

The system is designed to ensure full traceability of each recommendation, allowing linkage between input data, activated rules, and supporting literature sources, thereby enhancing interpretability and reproducibility.

While specific contents of the knowledge base and rule library remain proprietary, the system architecture, rule formalism, and data integration pipeline are fully described and reproducible at a conceptual level. The platform is designed to ensure consistency and reproducibility of outputs across similar input profiles and functions as a clinician-support tool rather than an autonomous decision-maker.

### 2.5. Personalized Microbiome Modulation Intervention

Following baseline biological and clinical assessment, each participant received an individualized microbiome modulation program generated by the AI-guided analytical platform. The intervention was designed to address patient-specific dysbiosis patterns identified through integrated interpretation of metagenomic microbiome data, inflammatory and nutritional biomarkers, clinical history, and patient-reported symptoms.

The resulting recommendations consisted of a combination of dietary modifications, targeted microbiome-directed supplementation, and lifestyle adjustments aimed at restoring microbial ecological balance and improving gastrointestinal function. All recommended products were commercially available, non-investigational supplements and nutritional interventions.

Examples of individualized recommendations included:

- Dietary adjustments aimed at increasing substrates for beneficial microbial metabolism (e.g., increased intake of fermentable fibers, resistant starch, or polyphenol-rich foods).
- Targeted probiotic or synbiotic supplementation, selected based on identified microbial depletion patterns (for example, supplementation strategies aimed at supporting Bifidobacterium or other short-chain fatty acid-producing taxa).
- Prebiotic compounds designed to promote microbial production of short-chain fatty acids such as butyrate.
- Micronutrient supplementation when baseline laboratory testing suggested deficiencies (e.g., vitamin D, B-complex vitamins).
- Lifestyle-related measures relevant to gastrointestinal function, including meal timing regularization, sleep optimization, and physical activity recommendations.

The specific combination of interventions varied between participants according to their baseline biological profile and symptom pattern. Participants were instructed to follow their individualized program continuously for three months while maintaining their usual oncologic care.

Due to the personalized nature of the intervention, no uniform protocol was applied across participants. Instead, each intervention represented an individualized combination of dietary, supplement-based, and lifestyle recommendations tailored to the specific biological profile of each patient.

### 2.6. Outcome Measures and Follow-Up Assessment

The study was designed to evaluate whether personalized microbiome modulation would translate into measurable clinical and biological improvement over a three-month period.

Primary outcome:

The primary endpoint was the change in daily stool frequency between baseline and Month 3, selected as a clinically relevant measure directly reflecting gastrointestinal function and patient quality of life.

Secondary outcomes:

Secondary outcomes included changes in self-reported energy levels, inflammatory and nutritional biomarkers, microbiome composition, and metabolic parameters. Gastrointestinal symptoms were assessed using structured questionnaires and daily stool diary entries, with particular attention to stool frequency, bowel pattern stability, and associated discomfort. In addition, participants reported their perceived energy levels using a simple numerical scale ranging from 1 to 10. This scale is not a formally validated instrument and has not been previously validated in this clinical context but was used as a pragmatic measure to capture patient-perceived functional status over time.

Blood biomarkers reflecting inflammatory and nutritional status were reassessed at Month 3. Microbiome sequencing was repeated to evaluate directional shifts in predefined bacterial taxa. Because microbial abundance was categorized using ordinal laboratory reference ranges, changes were analyzed as paired shifts in abundance category rather than purely quantitative fluctuations. This approach allowed us to assess whether microbiome modulation occurred in a biologically meaningful direction.

Exploratory outcomes:

Exploratory analyses were performed to examine the relationship between microbiome dynamics and clinical symptom response. Correlations between bacterial abundance changes and improvements in stool frequency or energy levels were evaluated, and multivariable models were constructed to explore independent predictors of symptom improvement. Clinical GI symptom responders were additionally characterized using a predefined reduction in daily stool frequency, enabling comparison of baseline biological features between responders and non-responders.

Baseline gastrointestinal symptom severity was heterogeneous, as inclusion was based on symptom persistence rather than standardized severity thresholds. In addition, the absence of validated gastrointestinal symptom scoring systems (e.g., GSRS or PRO-CTCAE) may limit comparability and affect the precision of symptom characterization across participants.

At Month 3, all baseline assessments were repeated, including microbiome sequencing, laboratory biomarkers, symptom questionnaires, and quality-of-life evaluation. This paired longitudinal design allowed evaluation of both symptom evolution and the coherence between microbial, metabolic, and clinical changes over time.

### 2.7. Statistical Analysis

All analyses were performed with the intention of evaluating intra-individual change over time and exploring the biological coherence between microbiome modulation and clinical symptom response. Statistical processing was conducted using IBM SPSS Statistics for Windows, Version 26.0 (IBM Corp., Armonk, NY, USA).

Because microbiome and biomarker data were provided as ordinal categories (very low to very high), these categories were converted into numerical ordinal scores ranging from 1 to 5 for statistical analysis. This approach reflects the reporting framework of the clinical microbiome platform used in this study and aligns with its application in a decision-support context, where microbial patterns are interpreted relative to reference ranges rather than absolute quantitative values. It enables standardized within-subject comparison of directional changes over time and facilitates integration with clinical interpretation.

However, this transformation reduces analytical resolution, may limit statistical power, and may reduce sensitivity to detect subtle microbial changes compared with fully quantitative metagenomic data. To mitigate these limitations, analyses focused on paired intra-individual changes over time, prioritizing directional consistency over absolute quantitative differences. Continuous variables were assessed for normality using the Shapiro–Wilk test. Normally distributed variables were analyzed using paired *t*-tests, whereas non-normally distributed or ordinal variables were analyzed using the Wilcoxon signed-rank test. Variables were summarized as mean ± standard deviation or median with interquartile range, as appropriate.

Between-group comparisons, specifically in exploratory analyses contrasting clinical GI symptom responders and non- GI symptom responders, were performed using the Mann–Whitney U test.

To explore potential relationships between microbiome dynamics and clinical improvement, correlations between changes in bacterial abundance and changes in clinical parameters were assessed using Spearman’s rank correlation coefficient. These analyses were designed to identify biologically meaningful associations rather than purely statistical co-variation.

Finally, multivariable linear regression models were constructed to examine independent predictors of clinical improvement. Baseline clinical parameters and microbiome shifts were entered as covariates to determine whether specific microbial changes remained significantly associated with symptom reduction after adjustment for baseline severity.

Where applicable, effect sizes and 95% confidence intervals were reported, particularly for multivariable regression analyses, to complement *p*-values and provide a more robust interpretation of the observed associations.

All statistical tests were two-tailed, and a *p*-value < 0.05 was considered statistically significant. Missing data were handled using multiple imputation techniques, with sensitivity analyses performed where appropriate.

Given the relatively small sample size and the exploratory nature of several analyses (including multiple biomarker comparisons, microbial taxa assessments, and correlation analyses), formal correction for multiple testing (e.g., Bonferroni or false discovery rate adjustment) was not systematically applied. Instead, these analyses were interpreted as exploratory and hypothesis-generating. Emphasis was placed on consistency of directional changes and biological coherence across findings rather than on isolated statistical significance. Therefore, the reported *p*-values should be interpreted with caution in the context of multiple parallel comparisons.

## 3. Results

The study included 29 female oncology patients, with a mean age in the late thirties and a relatively narrow age dispersion, reflecting a biologically active and professionally engaged population (Table 1). The relatively young age of the cohort reflects the predominance of breast and gynecologic malignancies among recruited participants. All participants had previously completed multimodal oncologic treatments, most commonly chemotherapy-based regimens, either alone or combined with radiotherapy, and in selected cases, immunotherapy or perioperative approaches. The spectrum of malignancies was heterogeneous, with breast cancer representing the largest subgroup, followed by gynecologic, gastrointestinal, thoracic, hematologic, and rare solid tumors. This diversity reflects real-world oncologic practice rather than a single-disease cohort, thereby increasing the ecological validity of the findings. Importantly, gastrointestinal symptoms were not transient. The median duration approached one year, with some patients reporting persistent manifestations extending beyond that. Most described chronic alterations in bowel habits, bloating, abdominal discomfort, and fluctuating stool frequency that had failed to respond to empirical symptomatic therapies prior to enrollment. All units and scales are specified within each table, with continuous variables expressed as mean ± standard deviation (SD) or median (interquartile range, IQR), as appropriate.

**Figure 1:**
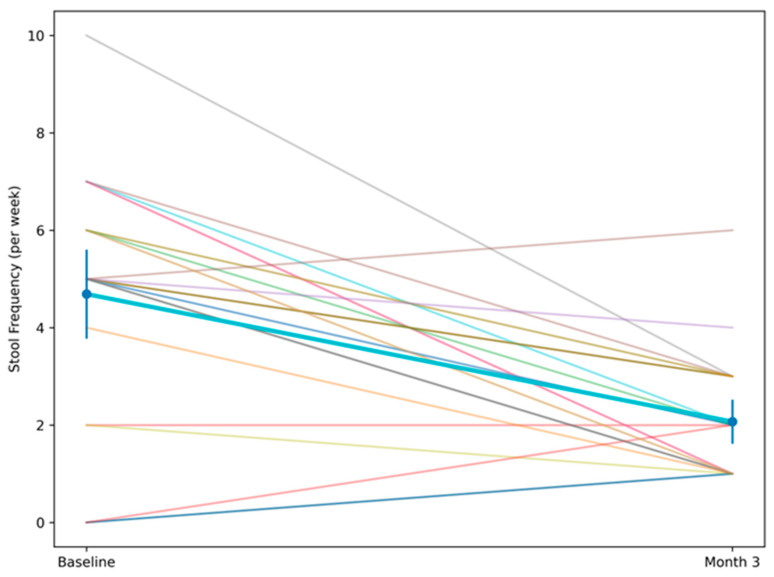
Individual trajectories and mean change in stool frequency (episodes/day) between baseline (M0) and Month 3 (M3) following AI-guided personalized microbiome modulation. Each line represents an individual patient. The bold line represents the cohort mean, and error bars indicate 95% confidence intervals.

After three months of personalized microbiome-targeted intervention, clinically meaningful improvements were observed (Table 2). Stool frequency decreased significantly at the cohort level, with a marked within-subject reduction. The magnitude of change exceeded expected day-to-day variability in bowel patterns, suggesting a consistent shift toward bowel pattern normalization. The paired analysis demonstrated high statistical significance, supporting that the change was unlikely to be due to random variation alone. In parallel, self-reported energy levels increased substantially. Patients frequently described this improvement qualitatively during follow-up visits as a “return of baseline vitality,” improved daily functionality, and better tolerance of routine activities. The magnitude of change was consistent across individuals and reached strong statistical significance. The individual trajectories of stool frequency over the three-month intervention period are illustrated in Figure 1. Most patients exhibited a consistent downward trend over time, mirroring the significant reduction observed at the cohort level and reflected in the decline of the cohort mean. The graphical representation highlights both consistency and inter-individual variability, which is expected in biologically complex systems such as the gut microbiome.

From a biological perspective, the intervention was associated with favorable modulation of short-chain fatty acid-related markers and micronutrient-associated pathways (Table 3). Butyrate-related parameters showed the most striking shift, with most participants moving toward higher, biologically favorable categories.

In contrast, acetate and propionate demonstrated more heterogeneous trajectories and did not show statistically robust cohort-wide shifts. GABA exhibited moderate directional improvement in a subset of participants but without reaching formal statistical significance. Lactate displayed a significant downward shift, reflecting normalization from higher toward lower abundance categories. Although this change is labeled as “worsened” in the ordinal framework due to category directionality, it represents a biologically favorable reduction consistent with improved metabolic balance. Vitamin-related biomarkers, particularly B-complex vitamins and vitamin D, showed consistent improvement trends. Vitamin B12 demonstrated one of the most pronounced upward shifts, followed by B6 and B3. These changes likely reflect both microbiome-driven metabolic restoration and personalized dietary optimization generated by the AI platform. Given the number of comparisons performed, these results should be interpreted as exploratory and not adjusted for multiple testing.

**Figure 2:**
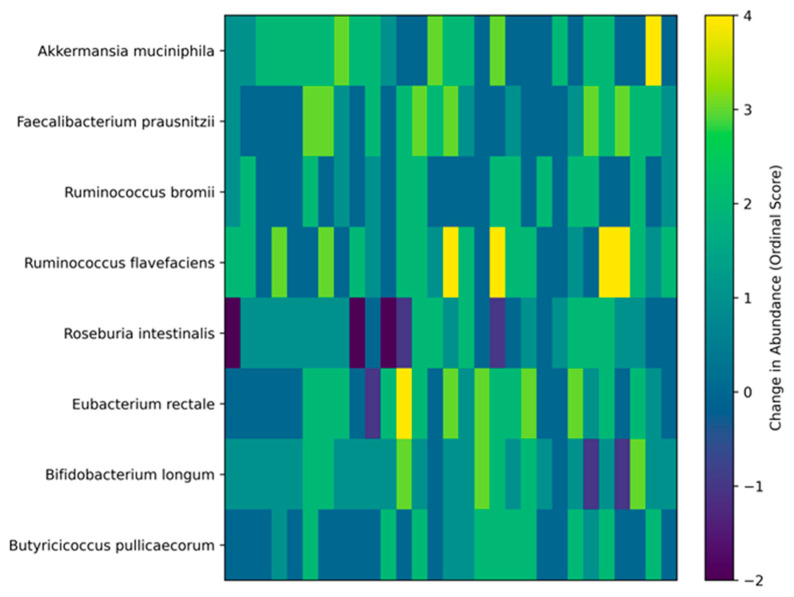
Heatmap of individual changes in key butyrate-producing bacterial species following AI-guided personalized microbiome modulation. Rows represent bacterial taxa and columns represent individual patients. Values reflect ordinal change scores (range −4 to +4), calculated as Month 3 minus baseline abundance categories (very low = 1 to very high = 5). Positive values indicate increased abundance, while negative values indicate decreased abundance. Color intensity reflects the magnitude and direction of change, with warmer colors (green to yellow) indicating increased abundance and cooler colors (blue to purple) indicating decreased abundance.

Targeted analysis of predefined, biologically relevant taxa revealed significant enrichment of several butyrate-producing and mucosal-supportive bacterial species after intervention (Table 4). *Akkermansia muciniphila* increased in a substantial proportion of participants, with no cases of decline observed. Similar upward shifts were observed for *Faecalibacterium prausnitzii*, *Ruminococcus flavefaciens*, and *Eubacterium rectale*. *Bifidobacterium longum* demonstrated one of the most consistent increases across the cohort. Notably, decreases were rare events across most taxa, suggesting that the intervention was not associated with destabilizing microbial shifts but rather with patterns of enrichment and normalization. The individual-level dynamics are visualized in Figure 2. The heatmap underscores that although the overall direction was consistent, the magnitude of change varied between individuals, supporting the premise that personalized modulation is more biologically appropriate than uniform supplementation strategies.

Correlation analysis (Table 5) explored whether microbiome shifts were associated with symptom improvement. While several taxa showed weak or non-significant associations, two signals emerged as biologically relevant. Increases in *Bifidobacterium longum* were significantly associated with reductions in stool frequency; however, this finding should be interpreted as exploratory. Additionally, increases in *Eubacterium rectale* and *Butyricicoccus pullicaecorum* demonstrated positive associations with improvements in energy levels, with Eubacterium rectale showing a statistically significant correlation.

Exploratory multivariable linear regression modeling was performed to further examine these associations (Table 6). Baseline stool frequency emerged as a strong independent predictor of magnitude of improvement; patients with more severe baseline disturbance experienced larger absolute reductions. Importantly, changes in *Bifidobacterium longum* remained independently associated with stool frequency improvement even after adjustment for other microbial variables. In contrast, changes in *Eubacterium rectale* and *Butyricicoccus pullicaecorum* did not retain independent predictive value in the multivariable model for stool frequency reduction.

When participants were stratified according to clinical symptom response (defined as a reduction of at least two stools per day), 21 patients met responder criteria (Table 7). Baseline *Bifidobacterium longum* levels differed significantly between GI symptom responders and non- GI symptom responders, suggesting a potential association with baseline stratification. In contrast, *Akkermansia muciniphila* did not significantly discriminate between groups at baseline.

**Figure 3:**
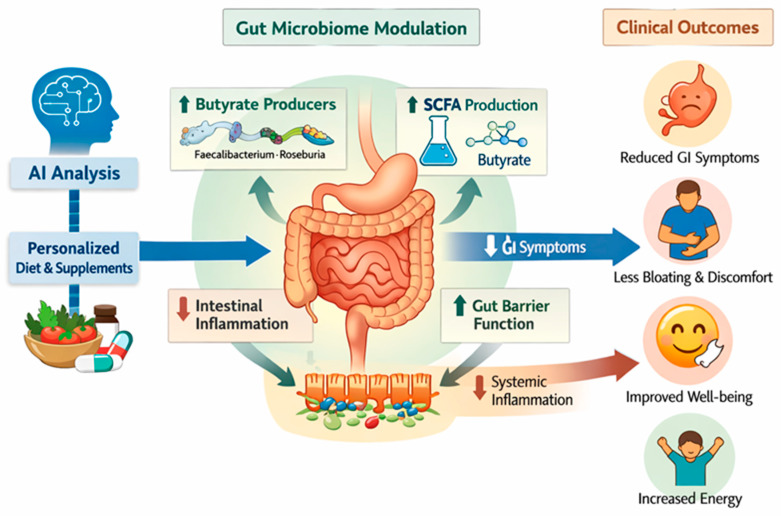
Systems-level pathway connecting personalized microbiome modulation to gastrointestinal symptom reduction and functional recovery. Proposed conceptual framework illustrating how AI-guided personalized microbiome modulation may be associated with clinical improvement in oncology patients with persistent gastrointestinal symptoms. Integration of microbiome profiling, clinical parameters, and biomarker data informs individualized dietary and supplement-based interventions, leading to increased abundance of butyrate-producing bacteria, enhanced short-chain fatty acid (SCFA) production, improved gut barrier integrity, and reduced intestinal and systemic inflammation. These biological changes are associated with decreased gastrointestinal symptom burden and improved overall well-being.

The systems-level pathway summarizing the proposed biological framework is presented in Figure 3. The integrated model suggests that AI-guided microbiome modulation may enhance butyrate-producing bacterial abundance, improve short-chain fatty acid balance, support epithelial barrier integrity, and attenuate low-grade inflammation. Clinically, these biological shifts were accompanied by reduced gastrointestinal symptom burden and improved functional energy status. While causality cannot be definitively established in this prospective validation design, the observed alignment between microbial, metabolic, and clinical changes supports the biological plausibility of these associations.

## 4. Discussion

Persistent gastrointestinal symptoms after oncologic treatment represent one of the most underestimated dimensions of survivorship care [15]. Although rarely life-threatening, chronic diarrhea, bloating, abdominal discomfort, and fluctuating bowel patterns profoundly affect quality of life, nutritional stability, and psychological recovery [16]. In this context, the present study sought to move beyond symptomatic management and to evaluate whether an AI-guided, personalized microbiome modulation strategy could be associated with clinically meaningful improvement in patients with persistent secondary GI dysfunction.

An important consideration when interpreting these findings relates to the inherent limitations of a single-arm study design. The observed clinical improvements may partially reflect non-specific effects, including regression to the mean, placebo responses, and behavioral modifications associated with study participation. Patients enrolled in structured interventional settings often demonstrate increased adherence to dietary recommendations, improved self-monitoring, and heightened health awareness, all of which may independently influence symptom evolution.

In addition, the Hawthorne effect, where individuals modify their behavior due to awareness of being observed, may have contributed to improved compliance with the personalized recommendations. While the magnitude and consistency of the observed clinical and biological changes are consistent with a biologically coherent signal, these non-specific influences cannot be fully excluded. Therefore, the findings should be interpreted as associative and hypothesis-generating rather than indicative of a definitive causal relationship. Importantly, the single-arm design does not allow disentangling the specific effect of the intervention from non-specific influences such as placebo response, spontaneous symptom fluctuation, or concurrent lifestyle changes. As a result, the observed improvements cannot be attributed exclusively to the AI-guided microbiome modulation strategy. This limitation is inherent to the study design and highlights the need for randomized controlled trials to confirm the causal contribution of the intervention.

The most immediate and clinically relevant finding was the significant reduction in stool frequency accompanied by a parallel improvement in self-reported energy levels. Importantly, these changes were not marginal. The magnitude of improvement is consistent with a meaningful shift in gastrointestinal stability rather than day-to-day fluctuation. In oncology practice, chronic post-treatment bowel dysfunction is often managed empirically with antidiarrheals, fiber modulation, or exclusion diets, yet durable normalization is uncommon [17]. Our findings therefore align with emerging evidence suggesting that treatment-related dysbiosis may persist long after cytotoxic or immunologic therapies have ended, contributing to sustained mucosal instability and altered motility [18]. Studies in cancer survivors have demonstrated reduced microbial diversity and depletion of short-chain fatty acid (SCFA)-producing taxa months to years after chemotherapy or radiotherapy, supporting the hypothesis that microbiome disruption may underlie chronic GI symptoms rather than merely accompany them [19,20].

From a biological standpoint, the most striking shift observed in our cohort was the robust increase in butyrate-related parameters and in several butyrate-producing bacterial taxa, including *Faecalibacterium prausnitzii*, *Roseburia intestinalis*, and *Eubacterium rectale*. The relevance of these findings extends beyond taxonomic curiosity. Butyrate is not simply a microbial metabolite; it is the primary energy substrate for colonocytes, a regulator of tight-junction protein expression, and a modulator of local immune responses through inhibition of NF-κB signaling and promotion of regulatory T-cell differentiation. Depletion of butyrate-producing species has been documented in inflammatory bowel disease, antibiotic-associated dysbiosis, and post-chemotherapy microbiome disruption [21,22,23]. The marked directional normalization observed in our study is consistent with a plausible mechanistic link between microbial restoration and symptom improvement.

The increase in *Akkermansia muciniphila* is consistent with this interpretation. This mucin-degrading bacterium has been associated with markers of improved epithelial barrier integrity and metabolic resilience. Experimental models suggest that *Akkermansia enhances* mucus layer thickness and reduces endotoxin translocation. In oncology populations, where mucosal injury may be induced by chemotherapy, pelvic radiotherapy, or repeated antibiotic exposure, restoration of barrier-supportive taxa may be associated with an important step toward functional recovery [24,25,26].

Interestingly, acetate and propionate did not demonstrate the same uniform shift as butyrate-related markers. This divergence may reflect the distinct metabolic roles of different SCFAs [27]. While acetate is more broadly produced and propionate participates in gluconeogenesis and hepatic signaling [27], butyrate exerts the most direct epithelial trophic effect [28]. The specificity of the butyrate signal in our cohort suggests that targeted ecological restoration, rather than global SCFA elevation, may represent an important determinant of symptom normalization.

The improvement in micronutrient-associated biomarkers, particularly B-complex vitamins and vitamin D, also warrants consideration. Chronic GI dysfunction in cancer survivors frequently is accompanied by subtle malabsorption, reduced dietary diversity, and low-grade inflammatory consumption of micronutrients [29]. The observed normalization may therefore be related to both improved absorptive function and restoration of microbial contributions to vitamin synthesis [30]. Certain Bifidobacterium species, including *Bifidobacterium longum*, are known to participate in B-vitamin metabolism [30,31]. The strong association between changes in *Bifidobacterium longum* and stool frequency reduction, including its persistence as an independent predictor in multivariable modeling, suggests that this taxon may be associated with a potentially relevant role in post-treatment gastrointestinal recovery; however, this observation should be interpreted as hypothesis-generating rather than indicative of a direct causal relationship.

The regression analysis provides additional insight. Baseline stool frequency emerged as a strong predictor of magnitude of improvement, indicating that patients with more severe dysfunction had greater room for recovery [32]. However, the independent association between *Bifidobacterium longum* change and stool reduction supports the concept that specific microbial shifts may be associated with symptom resolution, although the directionality of this relationship cannot be definitively established [33]. While causality cannot be definitively established in a single-arm design [34], the coherence between clinical, microbial, and metabolic changes strengthens biological plausibility. However, these findings should be interpreted with caution, given the limited sample size and the potential for model overfitting. Importantly, correlation does not imply causation, and the observed association may also reflect reverse causality, where improvement in gastrointestinal function creates a more favorable ecological niche for the expansion of specific microbial taxa such as *Bifidobacterium longum*.

It is also noteworthy that microbial changes were not uniform across individuals. The heatmap visualization demonstrates inter-individual variability in magnitude of taxonomic shifts, even when the overall direction was consistent [35]. This observation underscores a central premise of precision microbiome modulation: standardized probiotic or dietary interventions are unlikely to address the heterogeneity of post-oncologic dysbiosis [35]. Previous trials using empirical probiotic supplementation in oncology populations have produced mixed results [36], likely because interventions were not aligned with baseline microbial architecture [35]. By contrast, our AI-guided framework integrated microbiome data with clinical context to generate individualized recommendations [37], potentially explaining the consistency of symptomatic improvement despite taxonomic variability.

From a broader physiopathological perspective, these findings support the hypothesis that chronic post-treatment GI symptoms represent a state of sustained ecological imbalance rather than isolated motility disturbance. Cancer therapies can disrupt microbial communities, alter bile acid metabolism, reduce SCFA production, and impair epithelial integrity [38]. Persistent dysbiosis may perpetuate low-grade inflammation, increase intestinal permeability, and alter enteric nervous system signaling [39]. By restoring butyrate-producing and barrier-supportive taxa, the intervention may be associated with interruption of this self-sustaining cycle.

Clinically, the parallel improvement in stool frequency and perceived energy levels is particularly meaningful. Fatigue in cancer survivors is multifactorial, yet gut-derived inflammation and metabolic inefficiency may contribute [40]. SCFAs influence not only epithelial health but also systemic immune tone and mitochondrial function [27]. While speculative, the concordant rise in energy levels suggests that microbiome modulation may be associated with effects extending beyond local GI stabilization. However, this observation is based on a subjective, non-validated measure and should be interpreted with caution.

In the context of existing literature, our results align with emerging data linking microbiome composition to both oncologic treatment tolerance and systemic recovery [26,41]. Studies have shown that patients with higher abundance of butyrate-producing bacteria experience fewer treatment-related toxicities and improved immune regulation during immunotherapy. Although our study focused on survivorship rather than active treatment response, the underlying principle remains consistent: microbial ecology shapes host resilience.

The present findings suggest that AI-guided personalized microbiome modulation may represent a biologically coherent and clinically relevant approach to addressing persistent secondary gastrointestinal dysfunction in oncology patients [37]. The convergence of symptom reduction, metabolic normalization, and targeted microbial enrichment supports a systems-level interpretation in which ecological restoration is associated with functional recovery. However, the relatively short follow-up period limits conclusions regarding the long-term sustainability of these effects, and future studies should include extended longitudinal assessment to evaluate durability of both microbiome and clinical outcomes.

### Strengths, Limitations, and Future Directions

This study has several important strengths. First, it addresses a clinically relevant and often under-recognized unmet need in cancer survivorship care, namely the management of persistent gastrointestinal symptoms following oncologic treatment. Second, the integrative design represents a key innovation, combining microbiome profiling, inflammatory and nutritional biomarkers, and AI-guided personalization within a unified analytical framework. Third, the findings demonstrated consistent directional coherence across multiple domains, including clinical outcomes, microbial composition, and metabolic markers, supporting the internal validity of the observed patterns. Finally, the study reflects real-world clinical practice, as the intervention was applied in a heterogeneous oncology population without modification of standard oncologic care, enhancing the translational relevance of the results. Together, these strengths highlight the study as a real-world, hypothesis-generating model for precision supportive care in oncology.

At the same time, several limitations must be acknowledged. The absence of a control group represents the principal limitation, as the paired intra-individual design does not fully account for regression to the mean, placebo effects, or behavioral changes related to study participation. Consequently, causal inference cannot be established, and the findings should be interpreted as associative and hypothesis-generating.

The relatively small sample size (*n* = 29) represents an additional limitation. This limits statistical power, increases the risk of type II error, and restricts the stability and reliability of multivariable analyses, particularly given the number of predictors explored. In addition, the small cohort size constrains generalizability, as the findings may not be representative of broader and more heterogeneous oncology populations. As such, the results should be interpreted as exploratory rather than definitive, and larger studies are required to validate these findings and confirm the robustness of the observed clinical and microbiome-related associations.

Another important limitation relates to the assessment of clinical outcomes. Self-reported energy levels were measured using a simple numerical scale, which is inherently subjective and not formally validated. Furthermore, gastrointestinal symptoms were primarily evaluated using stool frequency and non-standardized questionnaires, without the use of validated quality-of-life or symptom-specific instruments (e.g., GSRS or PRO-CTCAE). This may limit the precision, reproducibility, comparability, and overall interpretability of the clinical outcome assessment, particularly in relation to the multidimensional nature of gastrointestinal symptom burden, including severity, discomfort, and quality-of-life impact. Consequently, the interpretation of symptom improvement should be approached with caution, and future studies should incorporate validated patient-reported outcome measures to strengthen clinical evaluation.

Microbiome assessment relied on ordinal abundance categories rather than fully quantitative metagenomic data, as raw sequencing outputs were not accessible. While this approach supports clinical interpretability, it reduces analytical resolution, may limit statistical power, and constrains the ability to detect subtle changes or accurately estimate the magnitude of biological effects. Therefore, findings should be interpreted primarily in terms of directional trends rather than precise quantitative differences.

The three-month follow-up period does not allow assessment of long-term durability of microbiome changes or sustained clinical improvement. Chronic survivorship-related gastrointestinal dysfunction may require longer-term ecological stabilization, and the persistence of both microbiome shifts and symptom improvement beyond the intervention window remains uncertain.

The partially proprietary nature of the AI platform represents an additional limitation, as it may restrict full methodological transparency and external reproducibility. Moreover, the intervention itself was inherently heterogeneous, combining dietary, supplementation, and lifestyle components tailored to individual profiles, making it difficult to isolate the contribution of specific elements. As a result, the intervention should be interpreted as a composite, systems-level approach rather than a standardized therapeutic protocol.

External independent validation by fully unaffiliated research groups remains limited at this stage, representing an important direction for future research. However, the system has been applied across multiple clinical cohorts and evaluated in peer-reviewed studies, including the present prospective validation study. Despite these constraints, the system architecture, rule formalism, and data integration pipeline are fully described and reproducible at a conceptual level, allowing independent implementation of comparable frameworks.

Given the number of parallel statistical comparisons performed across biomarkers, microbial taxa, and correlation analyses, no formal correction for multiple testing was applied. This increases the risk of type I error, and some statistically significant findings may represent false-positive associations. Therefore, results should be interpreted as exploratory, with emphasis on overall biological coherence rather than isolated statistical significance.

Finally, adherence to individual components of the intervention was based on patient-reported compliance and was not formally quantified using standardized adherence metrics, which may introduce variability in treatment exposure. Future studies should aim to disentangle the relative contribution of individual intervention components and incorporate objective adherence assessment strategies.

Future research should aim to validate these findings in more rigorous study designs. Randomized controlled trials are needed to distinguish specific intervention effects from non-specific behavioral and placebo-related influences. In addition, larger and more diverse cohorts, including both sexes and broader age ranges, are required to improve generalizability and to explore potential subgroup-specific responses. Future studies should also incorporate fully quantitative microbiome analyses alongside integrated metabolomic profiling to enhance resolution and enable more precise characterization of microbial–host interactions. Standardization of intervention components, as well as objective assessment of adherence, will be essential to further refine and validate personalized microbiome modulation strategies.

## 5. Conclusions

In this prospective validation study, AI-guided personalized microbiome modulation was associated with clinically meaningful intra-individual improvement in stool frequency and self-reported energy levels over a three-month period in a selected cohort of female oncology patients with persistent gastrointestinal symptoms.

These changes were accompanied by consistent directional shifts in butyrate-producing and barrier-supportive microbial taxa, as well as improvements in selected metabolic and micronutrient-related biomarkers, suggesting biologically coherent patterns across clinical and microbiome domains.

However, given the single-arm design, small sample size, use of non-validated symptom measures, and reliance on ordinal microbiome data, these findings should be interpreted as exploratory and hypothesis-generating rather than indicative of a causal effect.

Further validation in larger, more diverse cohorts using randomized controlled designs, quantitative microbiome analyses, and standardized clinical outcome measures is required to confirm the clinical utility and mechanistic relevance of this approach.

## Figures and Tables

**Table 1:** Baseline demographic and clinical characteristics of the study cohort.

Characteristic	Value
Number of participants	29
Age (years), mean ± SD	38.17 ± 4.83
Age range (years)	30–47
Sex: Female, *n* (%)	29 (100%)
Cancer Type, *n* (%)	
Breast cancer	9 (31.0%)
Cervical cancer	3 (10.3%)
Ovarian cancer	3 (10.3%)
Colorectal cancer	3 (10.3%)
Hodgkin lymphoma	2 (6.9%)
Endometrial cancer	2 (6.9%)
Lung cancer	2 (6.9%)
Gastric cancer	1 (3.4%)
Non-Hodgkin lymphoma	1 (3.4%)
Soft tissue sarcoma	1 (3.4%)
Glioblastoma	1 (3.4%)
Pancreatic cancer	1 (3.4%)
Treatment Modality, *n* (%)	
Chemotherapy alone	7 (24.1%)
Concomitant or sequential chemoradiotherapy	11 (37.9%)
Pelvic radiotherapy-based treatment	5 (17.2%)
Systemic therapy including immunotherapy	2 (6.9%)
Other chemotherapy regimens	4 (13.8%)
Duration of GI symptoms (months), median (IQR)	10 (5–13)
Duration of GI symptoms range (months)	0–18

Values are presented as mean ± standard deviation (SD), median (interquartile range, IQR), or number (percentage), as appropriate. Concomitant or sequential chemoradiotherapy includes both concurrent and sequential administration of chemotherapy and radiotherapy. Pelvic radiotherapy-based treatments were primarily administered in colorectal and gynecologic malignancies.

**Table 2:** Changes in clinical outcomes after three months of AI-guided personalized microbiome modulation.

Outcome	Baseline (Mean ± SD)	Month 3 (Mean ± SD)	Mean Change (Δ)	*p*-Value
Stool Frequency (episodes/day)	4.69 ± 2.41	2.07 ± 1.19	−2.62	<0.0001
Self-reported Energy Level (1–10)	4.00 ± 1.04	7.24 ± 1.12	+3.24	<0.0001

Within-subject comparison of clinical parameters at baseline (M0) and after three months of personalized microbiome modulation (M3). Continuous variables are presented as mean ± standard deviation (SD). Mean change (Δ) represents the difference between Month 3 and baseline values. Statistical significance was assessed using paired *t*-tests. A two-sided *p*-value < 0.05 was considered statistically significant.

**Table 3:** Changes in microbial metabolites and vitamin-related biomarkers from baseline to month 3.

Biomarker/Metabolite	Baseline Median (IQR) *	Month 3 Median (IQR) *	Improved *n* (%)	Unchanged *n* (%)	Worsened *n* (%)	*p*-Value (Wilcoxon)
Butyrate	1 (1–2)	3 (3–4)	28 (96.6%)	1 (3.4%)	0 (0.0%)	<0.0001
Acetate	2 (1–5)	3 (3–3)	14 (48.3%)	5 (17.2%)	10 (34.5%)	0.8597
Propionate	3 (2–3)	3 (3–3)	12 (41.4%)	8 (27.6%)	9 (31.0%)	0.3057
GABA	3 (2–3)	3 (3–4)	13 (44.8%)	9 (31.0%)	7 (24.1%)	0.2199
Lactate	5 (3–5)	3 (2–3)	4 (13.8%)	7 (24.1%)	18 (62.1%)	0.0017
Vitamin B3	2 (1–3)	3 (3–3)	21 (72.4%)	6 (20.7%)	2 (6.9%)	0.0004
Vitamin B6	2 (2–2)	3 (3–4)	22 (75.9%)	6 (20.7%)	1 (3.4%)	<0.0001
Vitamin B12	2 (1–3)	4 (3–5)	26 (89.7%)	3 (10.3%)	0 (0.0%)	<0.0001
Vitamin D	2 (1–3)	3 (2–3)	17 (58.6%)	8 (27.6%)	4 (13.8%)	0.0023

Because laboratory parameters were reported as ordinal categories (very low, low, normal, high, very high), these categories were converted into numerical ordinal scores ranging from 1 to 5 for statistical analysis. Median and interquartile range (IQR) therefore represent the distribution of these ordinal scores across participants. For certain biomarkers (e.g., lactate), decreases in ordinal category reflect biologically favorable changes despite being classified as “worsened” within the ordinal scoring system. * Median and interquartile range (IQR) are based on ordinal scores derived from laboratory-reported abundance categories (very low to very high), converted into numerical values (1–5) for statistical analysis. These values do not represent absolute quantitative concentrations.

**Table 4:** Changes in key bacterial species following AI-guided microbiome modulation.

Bacterial Species	Baseline Median (IQR) *	Month 3 Median (IQR) *	Increased *n* (%)	Unchanged *n* (%)	Decreased *n* (%)	*p*-Value (Wilcoxon)
***Akkermansia muciniphila***	1 (1–1)	3 (1–3)	19 (65.5%)	10 (34.5%)	0 (0.0%)	<0.0001
***Faecalibacterium prausnitzii***	1 (1–1)	3 (2–3)	18 (62.1%)	11 (37.9%)	0 (0.0%)	0.0002
***Ruminococcus bromii***	2 (1–3)	3 (2–3)	14 (48.3%)	15 (51.7%)	0 (0.0%)	0.0006
***Ruminococcus flavefaciens***	1 (1–2)	3 (3–3)	20 (69.0%)	9 (31.0%)	0 (0.0%)	<0.0001
***Roseburia intestinalis***	2 (2–3)	3 (3–3)	18 (62.1%)	6 (20.7%)	5 (17.2%)	0.0377
***Eubacterium rectale***	2 (1–3)	3 (3–3)	17 (58.6%)	11 (37.9%)	1 (3.4%)	0.0002
***Bifidobacterium longum***	2 (2–2)	3 (3–3)	25 (86.2%)	2 (6.9%)	2 (6.9%)	<0.0001
***Butyricicoccus pullicaecorum***	2 (1–3)	3 (3–3)	14 (48.3%)	15 (51.7%)	0 (0.0%)	0.0006

Abundance levels were recorded as ordinal categories (very low to very high). For statistical analysis, categories were converted into numerical ordinal scores (1–5). Median and interquartile range (IQR) therefore reflect the distribution of these ordinal scores across the study cohort. * Median and interquartile range (IQR) are based on ordinal scores derived from laboratory-reported abundance categories (very low to very high), converted into numerical values (1–5) for statistical analysis. These values do not represent absolute quantitative concentrations.

**Table 5:** Correlation between changes in bacterial species and clinical outcomes.

Bacterial Species (Δ)	ρ with Δ Stool Frequency	*p*-Value	ρ with Δ Energy	*p*-Value
***Akkermansia muciniphila***	0.005	0.9799	−0.050	0.7966
***Faecalibacterium prausnitzii***	0.034	0.8606	0.185	0.3357
***Ruminococcus bromii***	−0.102	0.5976	−0.128	0.5088
***Ruminococcus flavefaciens***	−0.009	0.9618	0.098	0.6140
***Roseburia intestinalis***	0.155	0.4218	0.120	0.5350
***Eubacterium rectale***	−0.237	0.2166	**0.478**	**0.0088**
***Bifidobacterium longum***	**−0.452**	**0.0138**	0.309	0.1030
***Butyricicoccus pullicaecorum***	−0.243	0.2032	**0.398**	**0.0325**

**Table 6:** Multivariable linear regression models for clinical improvement.

Predictor	β	95% CI	*p*-Value
Baseline stool frequency	**−0.787**	−0.978 to −0.597	**<0.0001**
*Δ Bifidobacterium longum*	**−0.783**	−1.344 to −0.222	**0.0082**
*Δ Eubacterium rectale*	0.155	−0.318 to 0.628	0.5050
*Δ Butyricicoccus pullicaecorum*	0.053	−0.528 to 0.633	0.8536

**Table 7:** Baseline characteristics according to clinical symptom response (Reduction ≥ 2 Stools/Day).

Baseline Variable	GI Symptom Responders (*n* = 21) Median Score *	Non- GI Symptom Responders (*n* = 8) Median Score *	*p*-Value
***Bifidobacterium longum***	2	2	**0.0128**
***Akkermansia muciniphila***	1	1	0.4047

Baseline biological parameters were compared between clinical GI symptom responders (defined as a reduction of ≥2 stools per day at Month 3) and non-GI symptom responders. Values are presented as median scores based on an ordinal scale * ranging from very low (1) to very high (5). Between-group comparisons were performed using the Mann–Whitney U test. A two-sided *p*-value < 0.05 was considered statistically significant.

## Data Availability

The datasets used and analyzed during the current study are available by request from the corresponding author.
